# In Vivo Imaging of Inflammation and Infection

**DOI:** 10.1155/2018/3817871

**Published:** 2018-05-17

**Authors:** Anne Roivainen, Xiang-Guo Li, Cristina Nanni, Weibo Cai, Sarah Ohrndorf

**Affiliations:** ^1^Turku PET Centre, University of Turku, 20521 Turku, Finland; ^2^Turku PET Centre, Turku University Hospital, 20521 Turku, Finland; ^3^Turku Center for Disease Modeling, University of Turku, 20521 Turku, Finland; ^4^Turku PET Centre, Åbo Akademi University, 20521 Turku, Finland; ^5^University of Bologna, Bologna, Italy; ^6^University of Wisconsin-Madison, Madison, WI, USA; ^7^Charité Universitätsmedizin, Berlin, Germany

Inflammation is a significant component of several chronic diseases involving the cardiovascular, metabolic, musculoskeletal, and nervous systems. Timely identification and localization of inflammation is critical for the adequate treatment of patients. Notably, also, infection always has inflammatory components. Molecular imaging such as positron emission tomography (PET) may reveal molecular and cellular changes and provide sensitive detection of inflammatory and/or infectious foci at an early stage of disease. In general, specific imaging of inflammatory processes may be a demanding task because of increased blood flow and enhanced vascular permeability causing unspecific uptake of imaging agents. Although several radiopharmaceuticals have been developed, no agent has been found with the optimal characteristics for imaging inflammation, and thus, there is still room for new, better agents and development of quantification methods.

This special issue consists of totally 11 articles (147 pages) including three review articles, six research articles, and two clinical studies. Nine articles are dealing with PET, one with magnetic resonance imaging (MRI), and one with PET-MRI. One of the research article deals with the modeling of PET, and the rest are preclinical/translational studies.

To perform diagnosis with PET, appropriate radiopharmaceuticals are needed. The radiopharmaceuticals are compounds possessing positron-emitting radionuclides, and gallium-68 (^68^Ga) is one of the most frequently used radionuclides in clinical PET. The review article “Prospective of ^68^Ga Radionuclide Contribution to the Development of Imaging Agents for Infection and Inflammation” by Velikyan provides an overview of ^68^Ga-labelled compounds for imaging of inflammation and infection in both preclinical and clinical settings. ^68^Ga can be conveniently obtained from commercially available generators, which facilitates the implementation of ^68^Ga-radiopharmaceutical research in many medical centers and in major human diseases including infection and inflammation.

In neuroinflammatory diseases such as multiple sclerosis (MS), there are clear clinical evidences for upregulation of the adenosine subtype 2A receptor (A_2A_). PET imaging of A_2A_ has become an approach to monitor functional changes and the treatment responses in the brain in the neuroinflammatory conditions. The review article “In Vivo PET Imaging of Adenosine 2A Receptors in Neuroinflammatory and Neurodegenerative Disease” by Vuorimaa et al. presents the progress in the research of PET imaging of A_2A_. So far, five PET ligands have entered into clinical trials, and each of them has their strengths and shortcomings regarding A_2A_ imaging.

Another important target for PET imaging of inflammation is 18 kDa translocator protein (TSPO). In the paper “TSPO PET Imaging: from Microglial Activation to Peripheral Sterile Inflammatory Diseases?” by Largeau et al., the authors discuss the exciting findings so far observed in the research of TSPO-targeted imaging. Furthermore, the challenges related to TSPO expression under physiological conditions and radioligand metabolism have been presented.

Fluorine-18 is a frequently used radionuclide for PET imaging, and its nonradioactive isotope fluorine-19 (^19^F) is very useful in MRI applications. In the work “Integrating a ^19^F MRI Tracer Agent into the Clinical Scale Manufacturing of a T-Cell Immunotherapy” by O'Hanlon et al., the authors report the scaling-up production of ^19^F-labelled T cells. The engineered T cells are originally used for immunotherapy. Upon incorporation of the cell sense agent into the T cells, it becomes possible to monitor the *in vivo* migration of the ^19^F-labelled T cells with MRI in the setting of treatment. Therefore, ^19^F-labelling is expected to have an added value in the development of cell-based therapeutics.

Osteomyelitis is an infectious bone disease, and PET imaging of osteomyelitis is often challenging, in particular in the cases of altered bone structures. In the work “A Comparative ^68^Ga-Citrate and ^68^Ga-Chloride PET/CT Imaging of *Staphylococcus aureus* Osteomyelitis in the Rat Tibia” by Lankinen et al., the authors have confirmed that ^68^Ga-citrate is a more appropriate radiopharmaceutical than ^68^Ga-chloride for PET imaging of osteomyelitis in their experimental settings in rats.

In addition to ^68^Ga-citrate, there are a number of other PET tracers that have been used for osteomyelitis imaging. In the work “Kinetic Modelling of Infection Tracers [^18^F]FDG, [^68^Ga]Ga-Citrate, [^11^C]Methionine, and [^11^C]Donepezil in a Porcine Osteomyelitis Model” by Jødal et al., with the aid of kinetic modelling, the authors conclude that [^18^F]FDG is an applicable tracer in general for PET imaging of osteomyelitis models in pigs.

In a clinical setting for osteomyelitis PET imaging, Salomäki et al. compare the imaging performance of ^68^Ga-citrate and [^18^F]FDG in their paper “Head-to-Head Comparison of ^68^Ga-Citrate and ^18^F-FDG PET/CT for Detection of Infectious Foci in Patients with *Staphylococcus aureus* Bacteraemia.” The results suggest that ^68^Ga-citrate and [^18^F]FDG have comparable performance in detection of osteomyelitis in general, but [^18^F]FDG shows higher intensity in soft tissues.

In another clinical study, Lankinen et al. use [^18^F]FDG to image osteomyelitis in their work “Intensity of ^18^F-FDG PET Uptake in Culture-Negative and Culture-Positive Cases of Chronic Osteomyelitis.” The results suggest that [^18^F]FDG can reveal osteomyelitis even in cases when microbiological culturing is still negative.

Regarding PET imaging of inflammation, vascular adhesion protein-1 (VAP-1) is one of the ideal targets. VAP-1 stays in its intracellular storage granules but rapidly relocates to endothelial cell surfaces upon inflammation. Virtanen et al. have studied two radiolabelled VAP-1 ligands in rats with skin inflammation and in mice with atherosclerosis, as described in their paper “Comparison of ^68^Ga-DOTA-Siglec-9 and ^18^F-Fluorodeoxyribose-Siglec-9: Inflammation Imaging and Radiation Dosimetry.”

In inflammation, folate receptors are often upregulated, which provides an alternative way for medical imaging and treatment. In the contribution “Imaging and Methotrexate Response Monitoring of Systemic Inflammation in Arthritic Rats Employing the Macrophage PET Tracer [^18^F]Fluoro-PEG-Folate” by Chandrupatla et al., the authors found out with the aid of ^18^F-labelled folate imaging that activated macrophages are reduced in the liver and spleen upon methotrexate treatment.

Crohn's disease is a chronic inflammatory disease that can affect the entire gastrointestinal track. To increase the accuracy in assessing Crohn's disease, Domachevsky et al. have added an apparent diffusion coefficient and a metabolic inflammatory volume to the magnetic resonance index of the activity score, and this has been described in their paper “Correlation of ^18^F-FDG PET/MRE Metrics with Inflammatory Biomarkers in Patients with Crohn's Disease: A Pilot Study.”

This special issue presents some new evidences of preclinical and clinical PET imaging of inflammation and infection, in addition to the overviews highly relevant to this field. We hope that this issue will provoke further research on these topics.

